# Fetal and Neonatal Alloimmune Thrombocytopenia in the Middle East: A Confirmed Case Highlighting Immunogenetic Diversity and Evidence-Based Neonatal Management

**DOI:** 10.7759/cureus.114213

**Published:** 2026-08-09

**Authors:** Hamza Haj Mohamad, Rand Soudan, Rosul Makkiyah, Sohyla Abdalla, Samiullah Haroon, Jennifer George, Abdelazeim Abdalla

**Affiliations:** 1 Pediatrics, Al Jalila Children's Speciality Hospital, Dubai, ARE; 2 Pediatrics and Neonatology, Mediclinic City Hospital, Dubai, ARE

**Keywords:** fnait, hpa incompatibility, human platelet antigen, middle east, neonatal alloimmune thrombocytopenia, neonatal thrombocytopenia

## Abstract

We report a confirmed case of fetal and neonatal alloimmune thrombocytopenia (FNAIT) in a preterm Arab neonate born to a mother with a previously affected pregnancy. Antenatal management consisted of weekly intravenous immunoglobulin initiated at 16 weeks of gestation. Following delivery, the infant developed severe thrombocytopenia with a nadir platelet count of 25 × 10⁹/L but showed no evidence of intracranial hemorrhage. Treatment with two platelet transfusions followed by intravenous immunoglobulin resulted in a rapid recovery of platelet counts and an uncomplicated clinical course. This case highlights successful evidence-based antenatal and neonatal management of recurrent FNAIT in a Middle Eastern family and adds to the limited regional literature on immunogenetically confirmed cases.

## Introduction

Fetal and neonatal alloimmune thrombocytopenia (FNAIT), also known as neonatal alloimmune thrombocytopenia (NAIT), is an immune-mediated disorder in which maternal IgG alloantibodies formed against fetal platelet antigens cross the placenta and cause fetal and neonatal platelet destruction, leading to thrombocytopenia and bleeding manifestations ranging from petechiae and ecchymoses to life-threatening hemorrhage, including intracranial hemorrhage (ICH) in utero or shortly after birth [1,2]. In contrast to hemolytic disease of the newborn, FNAIT may occur in a first pregnancy and is often first suspected when a clinically well neonate develops early, otherwise unexplained thrombocytopenia or bleeding in the setting of a mother with a normal platelet count [1,3]. Because ICH may occur antenatally in a substantial proportion of affected infants, the "fetal" component of FNAIT is clinically central and directly informs recurrence risk and preventive management in subsequent pregnancies [1,4].

The disease is driven by incompatibility in human platelet antigens (HPAs), which are polymorphic, largely diallelic antigens expressed on platelet membrane glycoproteins (notably integrins such as GPIIb/IIIa). Sensitization most commonly involves HPA-1a, although more than 34 HPAs have been described, and the standard nomenclature denotes each antigen system by number and allele (e.g., HPA-1a and HPA-1b) [1,5]. Epidemiologically, prospective studies and systematic reviews estimate FNAIT incidence broadly around approximately one in 1,000-3,000 live births, with severe neonatal thrombocytopenia being uncommon in the general newborn population and FNAIT representing a clinically important fraction of severe cases; among confirmed FNAIT cases, ICH remains the most feared complication [1,4]. Despite these estimates, there is no universally implemented screening program, and practice patterns remain heterogeneous across countries and centers, reflecting limitations in comparative trials and uncertainty around optimal risk stratification and treatment pathways [1,2].

Regionally, FNAIT appears underrecognized and underreported in the Middle East, and HPA immunogenetic distributions likely contribute to perceived rarity in Arab populations. Studies of HPA allele and genotype frequencies in Arab cohorts (e.g., Egyptian and Jordanian populations) demonstrate population-specific HPA distributions relevant to alloimmunization risk and donor availability, but robust regional epidemiologic incidence data and outcome registries remain limited [6,7]. This gap has practical implications: limited awareness, lack of organized screening, and constrained access to HPA-typed platelet products may delay diagnosis and complicate perinatal planning. Accordingly, case reporting from the region remains valuable to highlight clinical presentation across the fetal-neonatal spectrum, reinforce a structured diagnostic approach based on parental HPA typing and maternal antibody identification, and contextualize management strategies within current evidence-based recommendations and ongoing international efforts to harmonize care [1-3].

## Case presentation

A male newborn was admitted to the neonatal unit immediately after birth for evaluation and management of progressive thrombocytopenia. He was born at 35 weeks of gestation to a healthy gravida 2, para 2 mother by spontaneous vaginal delivery. Birth weight was 2235 grams (appropriate for gestational age). Apgar scores were 8 at one minute and 9 at five minutes. He was hemodynamically stable at birth.

This pregnancy was considered high risk for FNAIT due to a prior sibling affected by severe neonatal alloimmune thrombocytopenia at 36 weeks of gestation. The mother had no history of autoimmune disease or thrombocytopenia, and her platelet counts during pregnancy were within normal limits.

Maternal evaluation demonstrated homozygosity for HPA-1b. Antenatal testing identified maternal anti-HPA 2b/3a antibodies. Antenatal management consisted of intravenous immunoglobulin (IVIG) initiated at 16 weeks of gestation, administered weekly at a dose of 1 g/kg. Corticosteroids were not given except for near delivery to ensure lung maturation. Serial antenatal ultrasounds were performed to monitor fetal complications, and no evidence of intracranial hemorrhage was detected.

At birth, the infant's platelet count was 37 ×10⁹/L. He was clinically well, with no petechiae, bruising, or signs of active bleeding. Hemoglobin was 15.6 g/dL, and white blood cell count was 10.67 ×10⁹/L. Direct antiglobulin testing was negative. Serum bilirubin peaked at 140 µmol/L and was managed conservatively. Given the high-risk history and severe thrombocytopenia, platelet counts were monitored daily. A progressive decline in platelet count was observed, reaching a nadir of 25 ×10⁹/L on day 3 of life. A cranial ultrasound was performed and demonstrated no evidence of intracranial hemorrhage. In accordance with institutional protocol for suspected FNAIT, the infant received platelet transfusion on days 3 and 4 of life. Due to suboptimal platelet increment, high-dose IVIG at 1 g/kg per dose was administered on day 5 (two doses given). The platelet response and treatment timeline are summarized below in Table 1.

**Table 1:** Platelet trend and interventions. IVIG: intravenous immunoglobulin.

Day of life	Platelet count (×10⁹/L)	Intervention	Clinical notes
Day 0	37	—	Clinically stable
Day 1	53	—	No bleeding
Day 2	41	—	Downward trend noted
Day 3	25	Platelet transfusion #1	Treatment threshold reached
Day 4	36	Platelet transfusion #2	Suboptimal increment
Day 5	40	IVIG 1 g/kg (Dose 1)	Partial response noted and second-line treatment given
Day 6	67	IVIG 1 g/kg (Dose 2)	As advised by the hematologist
Day 8	234	Discharged	Stable platelet trend

Following the second transfusion and completion of IVIG therapy, the platelet count increased to 234 ×10⁹/L by day 8 of life and remained stable. The infant remained clinically well throughout admission, without evidence of bleeding. He was discharged on day 8 of life with outpatient hematology follow-up. He had two follow-ups; the first one was one week after discharge, where platelets were 388×10⁹/L, and the second follow-up was a week after that, where platelets were 450×10⁹/L.

Maternal serologic testing confirmed anti-HPA antibodies directed against paternal platelet antigens. Parental HPA typing demonstrated incompatibility consistent with the diagnosis of FNAIT. The mother’s platelet count remained normal, supporting an alloimmune rather than autoimmune mechanism.

## Discussion

Although FNAIT is globally estimated to occur in approximately one in 1,000-3,000 live births, epidemiologic data specific to Middle Eastern and Arab populations remain limited [2,3]. Most available incidence data derive from European screening studies, where anti-HPA-1a alloimmunization accounts for the majority of severe cases [8]. However, the distribution of HPAs varies by ethnicity and may influence regional disease patterns.

Recent immunogenetic studies from Saudi Arabia demonstrate HPA-1a and HPA-1b allele frequencies of approximately 0.831 and 0.169, respectively, with genotype frequencies of 75% HPA-1a/a and 25% HPA-1a/b among healthy donors [9]. Variability has also been observed in other antigen systems, including HPA-5 and HPA-15, suggesting that non-HPA-1-mediated incompatibilities may contribute to alloimmune risk in Arab and Iranian populations [10,11].

Additionally, the CD36-negative phenotype, reported in approximately 2.6-3.4% of individuals of Arabian origin, represents an alternative alloimmune mechanism capable of causing neonatal alloimmune thrombocytopenia and platelet transfusion refractoriness [12]. This pathway is distinct from classical HPA-1a-mediated disease and may be underrecognized in regional practice.

Despite these genetic data, there are no large-scale population-based incidence studies of FNAIT in the Middle East, and clinical registries remain lacking. Consequently, the true regional burden of disease is uncertain and likely underreported. Within this context, the present case provides clinically confirmed evidence of FNAIT in a purely Arab family with demonstrated HPA incompatibility, contributing to the limited outcome-based literature from the region.

FNAIT encompasses both fetal and neonatal disease, and clinical presentation differs significantly between these two periods. Antenatally, the most serious manifestation is ICH, which may occur in utero and can be the first indication of disease in an otherwise uncomplicated pregnancy. Neonatally, affected infants are typically well-appearing at birth but present with isolated thrombocytopenia of variable severity. Reported rates of ICH range from approximately 10% to 25% of confirmed cases, with a substantial proportion occurring antenatally; severe cases may result in long-term neurologic impairment or death [4,13,14].

As fetal bleeding may precede delivery, early recognition and structured antenatal surveillance are critical in pregnancies with a prior affected child. Postnatally, diagnosis relies on recognizing severe thrombocytopenia in an otherwise healthy infant and confirming parental HPA incompatibility with detection of maternal alloantibodies (Table 2) [1].

**Table 2:** Fetal vs. neonatal FNAIT: Clinical presentation, diagnosis, and management principles. Comparison of fetal and neonatal FNAIT: clinical presentation, diagnostic approach, and management principles. Data were synthesized and adapted from current international guidelines and key evidence reviews. Sources: Lieberman et al. (2019) [1], Pothof et al. (2025) [2], ACOG Practice Bulletin No. 207 (2019) [3], de Vos et al. (2022) [15], and Baker et al. (2019) [16]. * Detailed discussion of neonatal management thresholds and treatment controversies is provided in the following section. FNAIT: fetal and neonatal alloimmune thrombocytopenia; ICH: intracranial hemorrhage; HPA: human platelet antigen; IVIG: intravenous immunoglobulin; TORCH: toxoplasmosis, other infections, rubella, cytomegalovirus, and herpes simplex virus; DIC: disseminated intravascular coagulation; ITP: immune thrombocytopenia.

Domain	Fetal (FNAIT)	Neonatal (FNAIT)
Clinical presentation	• May be asymptomatic until bleeding • Intracranial hemorrhage (ventriculomegaly, increased head circumference, porencephalic cysts) • ICH may occur in utero and can warrant urgent delivery if detected on ultrasound	• Often clinically well at birth • Isolated thrombocytopenia • Petechiae, purpura, mucosal bleeding • Severe cases: intracranial hemorrhage (10-25% of cases) • Rarely fatal if untreated
Diagnostic approach	• Serial fetal ultrasounds to detect ICH • High-risk pregnancy labeling after first affected child • Avoid invasive fetal blood sampling in most cases • Parental HPA typing and maternal antibody testing	• Severe thrombocytopenia in otherwise healthy neonate • Rule out sepsis, TORCH, DIC, maternal ITP, preeclampsia • Normal Hb, WBC, coagulation profile • Direct antiglobulin test typically negative • Confirm by parental HPA typing and detection of maternal anti-HPA IgG
Management principles*	Guidelines for the fetal management in FNAIT are available from the American College of Obstetricians and Gynecologists: • Preventive strategy rather than invasive fetal therapy • Maternal IVIG 1-2 g/kg weekly from 16 to 20 weeks (earlier if prior ICH) • Serial ultrasounds • Avoid amniocentesis and invasive procedures • Steroids have no role in the management • Planned delivery (C-section preferred in prior ICH) • HPA-compatible platelets available at birth	There is a lack of guidelines for neonatal management in FNAIT • Immediate cranial ultrasound if platelets <50 ×10⁹/L • Serial platelet monitoring • Platelet transfusion: <25 ×10⁹/L if asymptomatic, <50 ×10⁹/L if bleeding/ill, or if major bleeding, prefer HPA-matched platelets; random donor acceptable • IVIG 1 g/kg (role debated; often given with transfusion) • Steroids have limited role

Current international consensus supports a platelet count threshold of 25 × 10⁹/L as the indication for platelet transfusion in asymptomatic neonates with FNAIT, with treatment recommended when the platelet count falls below this level even in the absence of bleeding [2,15]. Data from a large multicenter retrospective cohort demonstrated that platelet counts in untreated infants frequently rise spontaneously within the first week of life and that severe bleeding is uncommon in clinically stable infants with platelet counts above this threshold [15]. Systematic review data further indicate that while HPA-matched platelets produce superior post-transfusion increments, random donor platelets are generally sufficient to raise platelet counts above 30 × 10⁹/L, a level at which the risk of life-threatening hemorrhage is low [16]. For neonates with active bleeding or ICH, a higher transfusion threshold of 50 × 10⁹/L is recommended [2].

In treating neonatal alloimmune thrombocytopenia, HPA-compatible platelets are preferred because they are less susceptible to antibody-mediated destruction and produce significantly higher post-transfusion increments. In a large multicenter retrospective cohort, the median platelet increment after the first transfusion was 98 × 10⁹/L (IQR 67-134) with HPA-matched platelets compared with 59 × 10⁹/L (IQR = 35-94) for random donor platelets [15]. Earlier studies similarly reported increments ranging from 116 to 170 × 10⁹/L for matched platelets versus 27-68 × 10⁹/L for unmatched products [15]. Despite these differences in immediate increment, platelet counts by day 4 were not statistically different between groups (median = 125 × 10⁹/L vs. 87 × 10⁹/L, p = 0.15), suggesting convergence over time [15]. Importantly, random donor platelets remain clinically effective in urgent settings: in cohort analyses, 53% of neonates required transfusion, and random donor platelets raised platelet counts above 30 × 10⁹/L in nearly all cases, with counts remaining above this threshold for more than 24 hours in 71% of infants treated with random donor platelets alone; no major bleeding or ICH was observed following transfusion [15,17]. Washed maternal platelets, primarily used in intrauterine management, have also been shown to increase fetal platelet counts effectively; retrospective comparisons demonstrated similar clinical efficacy between maternal and HPA-matched donor platelets, although donor platelets achieved higher numerical increments [18]. Current expert consensus therefore recommends HPA-matched platelets when available, while recognizing that random donor platelets are a safe and acceptable alternative in the neonatal setting, particularly when compatible products are not immediately accessible [2].

IVIG is well established in antenatal management of pregnancies at risk for FNAIT, typically administered at 1 g/kg/week from 16 to 18 weeks of gestation until delivery to reduce the risk of fetal ICH and severe thrombocytopenia at birth [19,20]. In treated pregnancies, antenatal IVIG has been associated with favorable outcomes, with 82.6% of neonates achieving platelet counts ≥50 × 10⁹/L at birth (median = 172 × 10⁹/L) and no reported ICHs in one recent cohort [21]. In contrast, the postnatal role of IVIG remains uncertain, as platelet transfusion is consistently supported as first-line therapy for neonates with severe thrombocytopenia. In a multicenter cohort of 389 neonates, 28% received postnatal IVIG (alone or with platelets); however, platelet counts increased more rapidly after transfusion than after IVIG alone, and severe bleeding occurred in only 6% of cases, with most ICHs believed to have occurred antenatally [15]. A systematic review similarly concluded that adding IVIG to platelet transfusion did not improve platelet increments or clinical outcomes [16]. Additional cohort data demonstrated that random donor platelets alone were effective in correcting critically low counts, and adjunctive IVIG did not reduce transfusion requirements or enhance post-transfusion increments [17,22]. Collectively, current evidence supports antenatal IVIG as preventive therapy but indicates that postnatal IVIG does not confer additional benefit over platelet transfusion alone in terms of platelet increment or bleeding prevention, and its routine use in stable neonates remains unsupported by high-quality data.

Corticosteroids are not recommended as routine therapy in either antenatal or postnatal management of FNAIT. The most recent international Delphi consensus published in *The Lancet Haematology* advises against the routine use of antenatal steroids in HPA-1a-alloimmunized pregnancies with a previously affected child without ICH; their use may be considered only as adjunctive therapy in select high-risk pregnancies (e.g., prior fetal ICH), and even in this setting, consensus regarding benefit is lacking [2]. Randomized trials and the Cochrane systematic review comparing IVIG alone versus IVIG combined with corticosteroids (prednisone or dexamethasone) found no statistically significant differences in major outcomes, including fetal ICH, platelet count at birth, or preterm delivery [23]. Due to heterogeneity and limited sample sizes, definitive meta-analysis was not possible; however, available data do not support the routine addition of steroids to IVIG. In the postnatal setting, corticosteroids have no established role; platelet transfusion remains the primary therapy, and neither observational studies nor expert guidelines demonstrate benefit from neonatal steroid administration [1,24]. Overall, current evidence supports IVIG as the cornerstone of antenatal therapy and platelet transfusion as the cornerstone of postnatal management, with no demonstrated additive benefit of corticosteroids in either context.

Nipocalimab is a humanized monoclonal antibody targeting the neonatal Fc receptor (FcRn), the principal mediator of transplacental IgG transfer. By binding FcRn with high affinity, nipocalimab blocks IgG recycling and placental transport, thereby reducing maternal IgG levels and fetal exposure to pathogenic alloantibodies implicated in FNAIT [25,26]. Unlike IVIG, which acts as a competitive FcRn inhibitor, nipocalimab demonstrates more than 1000-fold higher FcRn-binding affinity and has been shown in related conditions to reduce maternal IgG levels by up to 85%, with corresponding reductions in cord blood IgG [27,28]. Its role in FNAIT remains investigational; ongoing phase III trials (FREESIA-1 and FREESIA-3) are evaluating weekly intravenous nipocalimab initiated at 13-18 weeks of gestation compared with standard therapy (IVIG with or without prednisone), with primary endpoints including severe fetal/neonatal bleeding, platelet count at birth, and transfusion requirements [28,29]. Nipocalimab is not yet approved for FNAIT, but its targeted mechanism offers a potential future alternative to IVIG pending confirmation of safety and efficacy in ongoing trials [30].

Universal screening for FNAIT is not currently implemented in most countries and remains controversial. Unlike RhD alloimmunization, no standardized population-based screening program exists, largely due to uncertainty regarding cost-effectiveness, variability in disease severity, and the absence of a prophylactic therapy comparable to anti-D immunoglobulin [1,2]. Northern European antenatal screening studies demonstrated that early identification of HPA-1a-negative females can reduce severe neonatal thrombocytopenia and ICH; however, these programs were conducted in well-resourced settings with centralized transfusion services [8]. Current international consensus supports targeted screening in high-resource environments but does not recommend universal implementation [2]. In the Middle East, where regional incidence data and outcome registries are limited, the feasibility of structured screening programs remains uncertain.

## Conclusions

In conclusion, FNAIT remains a potentially life-threatening but treatable condition requiring timely recognition and coordinated perinatal management. This case highlights confirmed FNAIT in a purely Arab family, emphasizing that clinically significant HPA incompatibility exists in Middle Eastern populations despite limited regional epidemiologic data. Current evidence supports platelet transfusion as the cornerstone of postnatal management, with HPA-compatible products preferred but random donor platelets effective when necessary; routine postnatal IVIG or corticosteroid use is not supported by high-quality evidence. Antenatal IVIG remains the standard preventive strategy in high-risk pregnancies, while emerging FcRn-targeted therapies may reshape future management. Increased regional awareness, structured registries, and evidence-based protocols are essential to optimize outcomes and inform screening considerations in diverse populations.

## References

[1] Lieberman L, Greinacher A, Murphy MF (2019). Fetal and neonatal alloimmune thrombocytopenia: recommendations for evidence-based practice, an international approach. Br J Haematol.

[2] Pothof R, van den Akker-van Marle EM, de Vos TW (2025). Rising to the challenge: an international Delphi consensus study on fetal and neonatal alloimmune thrombocytopenia. Lancet Haematol.

[3] American College of Obstetricians and Gynecologists (2019). ACOG Practice Bulletin No. 207: thrombocytopenia in pregnancy. Obstet Gynecol.

[4] Kamphuis MM, Paridaans NP, Porcelijn L, Lopriore E, Oepkes D (2014). Incidence and consequences of neonatal alloimmune thrombocytopenia: a systematic review. Pediatrics.

[5] Curtis BR (2015). Recent progress in understanding the pathogenesis of fetal and neonatal alloimmune thrombocytopenia. Br J Haematol.

[6] Husebekk A, El Ekiaby M, Gorgy G (2012). Foetal/neonatal alloimmune thrombocytopenia in Egypt; human platelet antigen genotype frequencies and antibody detection and follow-up in pregnancies. Transfus Apher Sci.

[7] Salem AH, Abdel Hamed AE, Abdalla EM, Almawi W (2014). Gene frequencies of human platelet alloantigens 1-5 in two Arab populations. Blood Transfus.

[8] Williamson LM, Hackett G, Rennie J (1998). The natural history of fetomaternal alloimmunization to the platelet-specific antigen HPA-1a (PlA1, Zwa) as determined by antenatal screening. Blood.

[9] Al-Ouda SK, Al-Banyan AA, Abdel Gader AG, Bayoumy NM, Al-Gahtani FH (2016). Gene frequency of human platelet alloantigens-1 to -6 and -15 in Saudi blood donors. Transfus Med.

[10] AlSuhaibani O, Chatti N, AlZahrani S (2025). Immunogenetic polymorphism of human platelet antigens in the Saudi population: insights into genetic diversity and alloimmune risk. Vox Sang.

[11] Kazemi MH, Malakootikhah F, Momeni-Varposhti Z, Falak R, Delbandi AA, Tajik N (2020). Human platelet antigen 1-6, 9 and 15 in the Iranian population: an anthropological genetic analysis. Sci Rep.

[12] Flesch BK, Scherer V, Opitz A, Ochmann O, Janson A, Steitz M, Zeiler T (2021). Platelet CD36 deficiency is present in 2.6% of Arabian individuals and can cause NAIT and platelet refractoriness. Transfusion.

[13] Ghevaert C, Campbell K, Walton J (2007). Management and outcome of 200 cases of fetomaternal alloimmune thrombocytopenia. Transfusion.

[14] Delbos F, Bertrand G, Croisille L, Ansart-Pirenne H, Bierling P, Kaplan C (2016). Fetal and neonatal alloimmune thrombocytopenia: Predictive factors of intracranial hemorrhage. Transfusion.

[15] de Vos TW, Winkelhorst D, Árnadóttir V (2022). Postnatal treatment for children with fetal and neonatal alloimmune thrombocytopenia: a multicentre, retrospective, cohort study. Lancet Haematol.

[16] Baker JM, Shehata N, Bussel J (2019). Postnatal intervention for the treatment of FNAIT: a systematic review. J Perinatol.

[17] Bakchoul T, Bassler D, Heckmann M (2014). Management of infants born with severe neonatal alloimmune thrombocytopenia: the role of platelet transfusions and intravenous immunoglobulin. Transfusion.

[18] Giers G, Wenzel F, Fischer J, Stockschläder M, Riethmacher R, Lorenz H, Tutschek B (2010). Retrospective comparison of maternal vs. HPA-matched donor platelets for treatment of fetal alloimmune thrombocytopenia. Vox Sang.

[19] Winkelhorst D, Murphy MF, Greinacher A (2017). Antenatal management in fetal and neonatal alloimmune thrombocytopenia: a systematic review. Blood.

[20] Regan F, Lees CC, Jones B, Nicolaides KH, Wimalasundera RC, Mijovic A (2019). Prenatal management of pregnancies at risk of fetal neonatal alloimmune thrombocytopenia (FNAIT): scientific impact paper no. 61. BJOG.

[21] Zloto K, Cohen S, Batsry L (2025). Effectiveness of antenatal intravenous immunoglobulin treatment in recurrent fetal and neonatal alloimmune thrombocytopenia. Ultrasound Obstet Gynecol.

[22] te Pas AB, Lopriore E, van den Akker ES, Oepkes D, Kanhai HH, Brand A, Walther FJ (2007). Postnatal management of fetal and neonatal alloimmune thrombocytopenia: the role of matched platelet transfusion and IVIG. Eur J Pediatr.

[23] Rayment R, Brunskill SJ, Soothill PW, Roberts DJ, Bussel JB, Murphy MF (2011). Antenatal interventions for fetomaternal alloimmune thrombocytopenia. Cochrane Database Syst Rev.

[24] Bertrand G, Kaplan C (2014). How do we treat fetal and neonatal alloimmune thrombocytopenia?. Transfusion.

[25] Seth NP, Xu R, DuPrie M (2025). Nipocalimab, an immunoselective FcRn blocker that lowers IgG and has unique molecular properties. MAbs.

[26] Moise KJ Jr, Oepkes D, Lopriore E, Bredius RG (2022). Targeting neonatal Fc receptor: potential clinical applications in pregnancy. Ultrasound Obstet Gynecol.

[27] Moise KJ Jr, Ling LE, Oepkes D (2024). Nipocalimab in early-onset severe hemolytic disease of the fetus and newborn. N Engl J Med.

[28] Bussel JB, Cines DB, Blumberg RS (2025). Neonatal Fc receptor - biology and therapeutics. N Engl J Med.

[29] Tiller H, Tiblad E, Baker P, Van Valkenburgh H, Heerwegh D, Keshinro B (2026). Design of a phase 3, multicenter, randomized, placebo-controlled, double-blind study of nipocalimab in pregnancies at risk for fetal and neonatal alloimmune thrombocytopenia. Am J Perinatol.

[30] Bussel J, Stegmann B, Baker P (2026). Design of a phase 3, multicenter, randomized, open-label study of nipocalimab or IVIG and prednisone in pregnancies at risk for fetal and neonatal alloimmune thrombocytopenia. Am J Perinatol.

